# miR‐181a and miR‐150 regulate dendritic cell immune inflammatory responses and cardiomyocyte apoptosis *via* targeting JAK1–STAT1/c‐Fos pathway

**DOI:** 10.1111/jcmm.13201

**Published:** 2017-06-09

**Authors:** Jianbing Zhu, Kang Yao, Junjie Guo, Hongtao Shi, Leilei Ma, Qian Wang, Haibo Liu, Wei Gao, Aijun Sun, Yunzeng Zou, Junbo Ge

**Affiliations:** ^1^ Shanghai Institute of Cardiovascular Diseases Zhongshan Hospital Fudan University Shanghai China; ^2^ Department of Cardiology The Affiliated Hospital of Qingdao University Qingdao Shandong China; ^3^ Department of Laboratory Medicine Shanghai Chest Hospital affiliated to Shanghai Jiaotong University Shanghai China

**Keywords:** dendritic cells, immune inflammatory response, microRNAs, cardiomyocyte apoptosis

## Abstract

The immune inflammatory response plays a crucial role in many cardiac pathophysiological processes, including ischaemic cardiac injury and the post‐infarction repair process. MicroRNAs (miRNAs) regulate the development and function of dendritic cells (DCs), which are key players in the initiation and regulation of immune responses; however, the underlying regulatory mechanisms remain unclear. Here, we used the supernatants of necrotic primary cardiomyocytes (Necrotic‐S) to mimic the myocardial infarction (MI) microenvironment to investigate the role of miRNAs in the regulation of DC‐mediated inflammatory responses. Our results showed that Necrotic‐S up‐regulated the DC maturation markers CD40, CD83 and CD86 and increased the production of inflammatory cytokines, concomitant with the up‐regulation of miR‐181a and down‐regulation of miR‐150. Necrotic‐S stimulation activated the JAK/STAT pathway and promoted the nuclear translocation of c‐Fos and NF‐κB p65, and silencing of STAT1 or c‐Fos suppressed Necrotic‐S‐induced DC maturation and inflammatory cytokine production. The effects of Necrotic‐S on DC maturation and inflammatory responses, its activation of the JAK/STAT pathway and the induction of cardiomyocyte apoptosis under conditions of hypoxia were suppressed by miR‐181a or miR‐150 overexpression. Taken together, these data indicate that miR‐181a and miR‐150 attenuate DC immune inflammatory responses *via* JAK1–STAT1/c‐Fos signalling and protect cardiomyocytes from cell death under conditions of hypoxia.

## Introduction

MI, which is the leading cause of death in most industrialized nations, is characterized by cardiomyocyte death caused by sudden loss of oxygen and nutrient supply to the heart 1. Ischaemic injury induces an acute inflammatory response that plays a role in the repair process and is characterized by the recruitment of neutrophils to the ischaemic tissue and the production of inflammatory mediators, reactive oxygen species and proteases, followed by the mobilization of monocytes 2, 3, 4. CD4^+^ T cells, which become activated after MI, play an important role in myocardial wound healing 5. Studies showed that dying cells produced by pressure disruption or hypoxic injury are capable of activating the innate system and inducing a sterile inflammatory response 6, 7. Necrotic but not apoptotic cell death releases cell constituents (such as heat shock proteins and high‐mobility group B1 protein), which can trigger DC maturation 8, 9.

DCs are antigen‐presenting cells that develop directly from myeloid progenitors in the bone marrow and circulating blood monocytes and initiate primary T cell responses, linking innate and adaptive immune responses 10. Stimulation by inflammatory signals or uptake of pathogenic antigens leads to the migration of immature DCs to secondary lymph organs, where they undergo a maturation process involving the secretion of cytokines and the up‐regulation of surface major histocompatibility complex (MHC) II and costimulatory molecules such as CD80, CD86 and CD40 11. The maturation of DCs is characterized by enhanced differentiation of effector CD4^+^ T cells from naive T cell precursors 12, 13. DCs play an essential role in the modulation of immune responses, underscoring the importance of identifying regulatory factors and mechanisms involved in DC maturation and function.

MicroRNAs (miRNAs), which are small noncoding RNAs that bind to the 3′ untranslated region (3′‐UTR) of target mRNAs to modulate gene expression, have been implicated in the regulation of the immune function of DCs and the pathogenesis of atherosclerosis, the main cause of coronary heart disease 14. Several miRNAs play a role in the regulation of immune responses such as the differentiation of B and T cells, the proliferation of monocytes and neutrophils and the release of inflammatory cytokines 15. In addition, many miRNAs have been identified that are involved in DCs at various stages, including differentiation from haematopoetic precursor, steady‐state or immature stage DCs, and during DC maturation 16.

In our previous study, we identified c‐Fos as a target of miR‐181a and showed that miR‐181a negatively regulates the oxidized‐LDL (ox‐LDL)‐induced immune inflammatory response in DCs by down‐regulating c‐Fos 12. Here, we examined the role of miR‐181a and miR‐150 in the modulation of DC inflammatory responses and cardiomyocyte apoptosis in the context of the post‐MI microenvironment induced by the supernatants of necrotic primary cardiomyocytes (Necrotic‐S).

## Materials and methods

### Culture of bone marrow dendritic cells

Bone marrow dendritic cells (BMDCs) were isolated from C57BL/6 mice as described previously 12. BMDCs were cultured in complete RPMI‐1640 medium (Invitrogen, Carlsbad, CA, USA) containing 10% FBS (Invitrogen) and supplemented with 20 ng/ml recombinant mouse granulocyte–macrophage colony‐stimulating factor and 10 ng/ml IL‐4 (R&D Systems, Minneapolis, MN, USA). After 48 hrs, non‐adherent cells were washed off and the remaining clusters were cultured; the medium was changed every 48 hrs. On day 7, the cells were collected for treatment with supernatants from necrotic primary cardiomyocytes or LPS or transfection. Mouse‐specific STAT1, c‐Fos and control siRNA were purchased from Santa Cruz Biotechnology (Santa Cruz, CA, USA). The phenotypes of BMDCs were determined using antimouse CD11c, CD40, CD80, CD83, CD86 and MHC‐II antibodies (BD Pharmingen, San Diego, CA, USA).

### Primary cardiomyocyte culture

Primary cardiomyocytes were prepared from neonatal C57BL/6 mice (1–3 days old) as described previously 17. Briefly, hearts were excised and placed in ice‐cold Hanks’ solution that contains in mM: NaCl 136, KCl 4.2, dextrose 5.6, KH_2_PO_4_ 0.44, NaH_2_PO_4_ 0.34, NaHCO_3_ 4.2, HEPES (pH 7.4) 5 and 100 U/ml penicillin–streptomycin (Invitrogen). These hearts were minced and digested using collagenase for 2 hrs. The suspended cells were pelleted by centrifugation at 800 *g* for 5 min., and the cell pellet was resuspended in HG/DMEM medium containing 10% FBS and 1% (v/v) streptomycin/penicillin. Non‐cardiomyocytes were removed by differential attachment and enriched myocytes cultured in HG/DMEM medium containing 10% FBS and 1% (v/v) streptomycin/penicillin at 5% CO_2_ atmosphere at 37°C. As previously described 7, 18, necrosis of cells was induced by three cycles of freezing in a dry ice/ethanol bath followed by immediate thawing at 37°C, confirmed by flow cytometry analysis with annexin V and propidium iodide (PI) staining. Necrotic cells were positively stained with annexin V and PI up to 95% (Fig. S1). Supernatants were prepared from necrotic cells (5 × 10^5^/ml) by centrifugation 16760 × g for 10 min. at 4°C.

During hypoxia culture, co‐cultured BMDCs and cardiomyocytes were exposed to the hypoxia condition (2% O_2_, 5% CO_2_, 93% N_2_) in an oxygen‐control incubator (Thermo Fisher Scientific Inc, Waltham, MA, USA) for 24 hrs.

### BMDC analysis by flow cytometry

Bone marrow dendritic cells were analysed by flow cytometry for surface marker expression using antibodies against CD40, CD80, CD83, CD86 and MHC‐II (BD Pharmingen).

### Lentiviral vector transductions of miRNA precursors or inhibitors

This study used miExpress^™^ miRNA precursors for mmu‐miR‐181a, mmu‐miR‐150 and a scrambled control; miArrest^™^ miRNA inhibitors for mmu‐miR‐181a, mmu‐miR‐150 and a scrambled control; and Lenti‐Pac^™^ HIV expression packaging kit (GeneCopoeia, Rockville, MD, USA). Recombinant lentiviruses were produced by cotransfecting 293T cells with the lentivirus expression plasmid and packaging plasmids according to the manufacturer's protocol (GeneCopoeia). Infectious lentiviruses were harvested at 48 hrs post‐transfection, centrifuged to eliminate cell debris and then filtered through 0.45‐μm polyethersulfone (PES) low protein‐binding filters. Infectious titre was determined by fluorescence‐activated cell sorting analysis of GFP in 293T cells. Virus titres were in the range of 10^8^ transducing units/ml medium.

Bone marrow dendritic cells were plated at a density of 2 × 10^4^ cells per well in a 24‐well plate. After 24 hrs, the cells were transduced with 0.5 ml of recombinant lentiviral suspension in complete medium with polybrene at a final concentration of 8 μg/ml at 37°C and 5% CO_2_. After 24 hrs of transfection, the medium was replaced with fresh complete medium, and reporter gene expression was examined by fluorescence microscopy and flow cytometry after 5–6 days.

### RNA extraction and miRNA real‐time PCR

Cells were washed with ice‐cold PBS, and total RNA was isolated using the TRIzol reagent (Invitrogen) according to the manufacturer's protocol. cDNA was synthesized using One Step PrimeScript miRNA cDNA Synthesis kit (TaKaRa, Dalian, China). miRNA‐specific primers were designed according to the manufacturer's instructions and synthesized at Applied Biosystems (Foster City, CA, USA). U6 RNA was used as endogenous controls. Real‐time PCR was performed using SYBR RT‐PCR kit (TakaRa) and monitored by a 7500 Real‐Time PCR System (Applied Biosystems). Samples were analysed in triplicate. The level of miRNA expression was measured according to the 2^−ΔΔCt^ method.

### Splenic CD4^+^ T cell culture and Proliferation assay

Splenic CD4^+^ T cells were isolated using magnetic‐activated cell sorting (MACS; Miltenyi Biotec, Bergisch Gladbach, Germany) according to the manufacturer's protocol. Briefly, positive selection of CD4^+^ T cells from 8‐week‐old C57BL/6 mouse spleens was performed according to the manufacturer's protocol using CD4 Microbeads (Miltenyi Biotec). CD4^+^ T cells (2 × 10^5^ cells/well), stimulated with phytohemagglutinin‐M (PHA‐M) (5 μg/ml, Sigma‐Aldrich, St. Louis, MO, USA), were co‐cultured with allogeneic BMDCs (2 × 10^4^ cells/well) in complete medium. For T cell proliferation assay, BMDCs were stimulated with Necrotic‐S or transfected with miRNA precursors or inhibitors. T cell proliferation was determined by thymidine incorporation on days 2, 5 and 7. To determine cell proliferation, cells were pulsed with [^3^H] thymidine during the last 18 hrs of culture and incorporated radioactivity was quantified using a liquid scintillation counter (Beckman, Fullerton, CA, USA).

### Western blot analysis

Cells were lysed, and the total, cytosolic or nuclear protein was extracted. Protein concentration was estimated with a BCA assay reagent (Pierce, Rockfold, IL, USA). Protein samples were separated by SDS‐PAGE, transferred to PVDF membranes and immunoblotted with rabbit–antimouse polyclonal antibodies against p‐JAK1, JAK1, p‐STAT1, STAT1, c‐Fos, HSF‐1, p‐65, Cox‐4, Bcl‐2, Bax, p‐STAT3, STAT3, p‐STAT5, STAT5 (Santa Cruz Biotechnology), H2A (Cell Signaling Technologies, Beverly, MA, USA), active caspase‐3 or β‐actin (Abcam, Cambridge, MA, USA). The proteins were visualized using an enhanced chemiluminescent detection system (Pierce).

### Immunofluorescence staining

Cells were treated with supernatants from necrotic primary cardiomyocytes or transfection, fixed with 4% paraformaldehyde, permeabilized in ice‐cold ethanol or methanol, and treated with blocking buffer (PBS/0.1% Tween‐20/2% FCS) before immunostaining. Cells were incubated for 30 min. at room temperature with anti STAT1 or c‐Fos followed by the goat anti‐rabbit Cy3 or donkey anti‐rabbit Alexa Fluor 488R‐conjugated secondary antibodies (Invitrogen), respectively. Cells were washed in PBS/0.1% Tween‐20, and slides were mounted with Vectashield‐R containing DAPI for nuclear staining (Vector Laboratories, Burlingame, CA, USA). Cells were visualized with a Zeiss Imager M2 Apotome fluorescence microscope.

### Luciferase reporter assays

HEK293T cells (2 × 10^5^ cells per well) were plated in 24‐well plates with DMEM supplemented with 10% foetal bovine serum and 1% penicillin/streptomycin 1 day before transfection. The wild‐type (WT) and a mutant (MUT) 3′ UTR of STAT1 or c‐Fos were cloned into the pGL3‐control vector (Promega, Shanghai, China) to generate the reporter plasmids. The pRL‐CMV vector (Promega) was used as an internal control. 293T cells were cotransfected with the reporter plasmid containing either WT or MUT STAT1 or c‐Fos 3′ UTR, and miR‐181a‐pre or miR‐150‐pre, or control‐pre using Lipofectamine 2000 (Invitrogen). Cells were harvested 24 hrs later, and the relative luciferase activity was measured with the Dual‐Luciferase Reporter Assay System (Promega).

### Apoptosis analysis by TUNEL

Terminal deoxynucleotidyl transferase‐mediated dUTP nick end labelling (TUNEL) staining was performed using the ApopTag fluorescein *in situ* apoptosis detection kit (Chemicon, Temecula, CA, USA) according to the manufacturer's instructions. The binding of fluorescent antibodies in positive cells was determined under a fluorescent microscope.

### Statistical analysis

Data are represented as mean ± standard deviation (S.D.). Differences between groups were compared by Student's *t*‐test. Multiple comparisons were made using a one‐way analysis of variance followed by Fisher's tests. *P* < 0.05 was considered to be statistically significant. All assays were performed at least three times.

## Results

### The supernatants of necrotic primary cardiomyocytes promoted DC maturation

**Figure 1:**
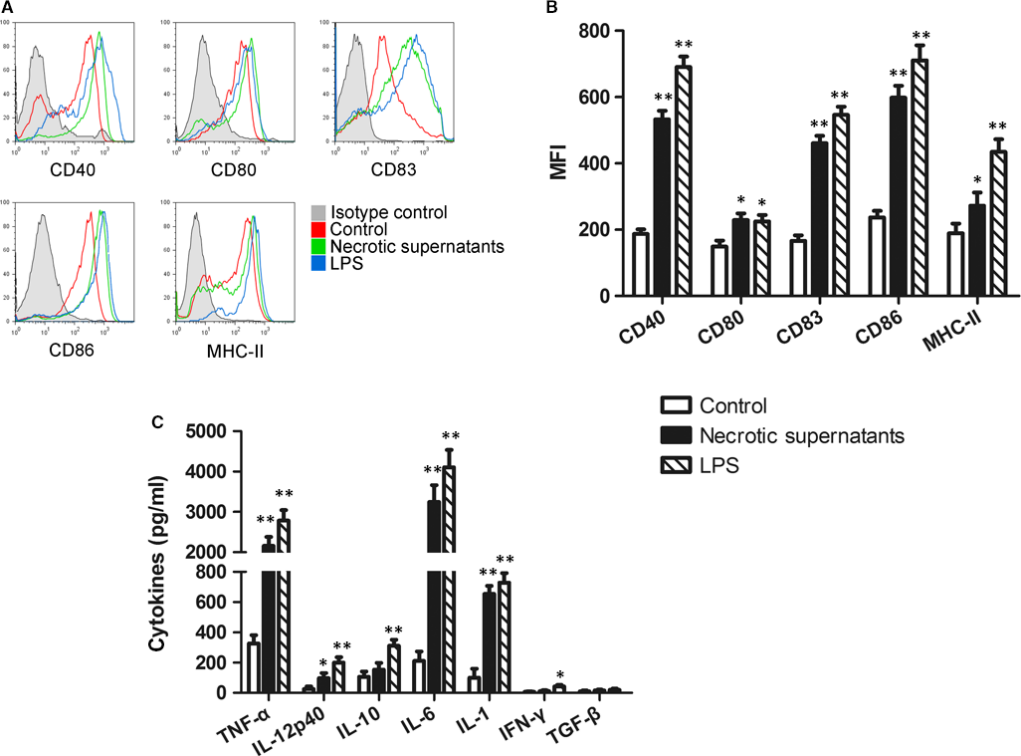
The supernatants of necrotic primary cardiomyocytes promote DC maturation. Bone marrow‐derived DCs (BMDCs) were either unstimulated (control group) or stimulated with the supernatants of necrotic primary cardiomyocytes (Necrotic‐S) or LPS (positive control) for 24 hrs. The expression levels of CD40, CD80, CD83, CD86 and MHC‐II were determined by flow cytometry. All data shown were gated on CD11c+ cells. (**A**) The histogram was obtained from a single experiment that is representative of three independent experiments. (**B**) The changes in mean fluorescence intensity (MFI) of CD40, CD80, CD86 and MHC‐II in the spleen CD11c+ cells were analysed. (**C**) Expression of cytokines including TNF‐α, IL‐12p40, IL‐10, IL‐6, IL‐1, INF‐γ and TGF‐β were measured by ELISA. The values represent the mean (S.D.) from three independent experiments. *P < 0.05, **P < 0.01, *versus* the Control group.

The supernatants of necrotic primary cardiomyocytes (Necrotic‐S) were used to mimic the MI microenvironment. Cultured BMDCs were either unstimulated (control group) or stimulated with the Necrotic‐S or LPS (positive control group) for 24 hrs, and analysed by flow cytometry. The results showed that Necrotic‐S up‐regulated the expression of the DC maturation markers CD40, CD80, CD83, CD86 and MHC‐II (Fig. 1A and B), and increased the levels of inflammatory cytokines, including TNF‐α, IL‐12p40, IL‐6 and IL‐1 (Fig. 1C).

### miR‐181a and miR‐150 regulate Necrotic‐S‐induced DC inflammatory response

**Figure 2:**
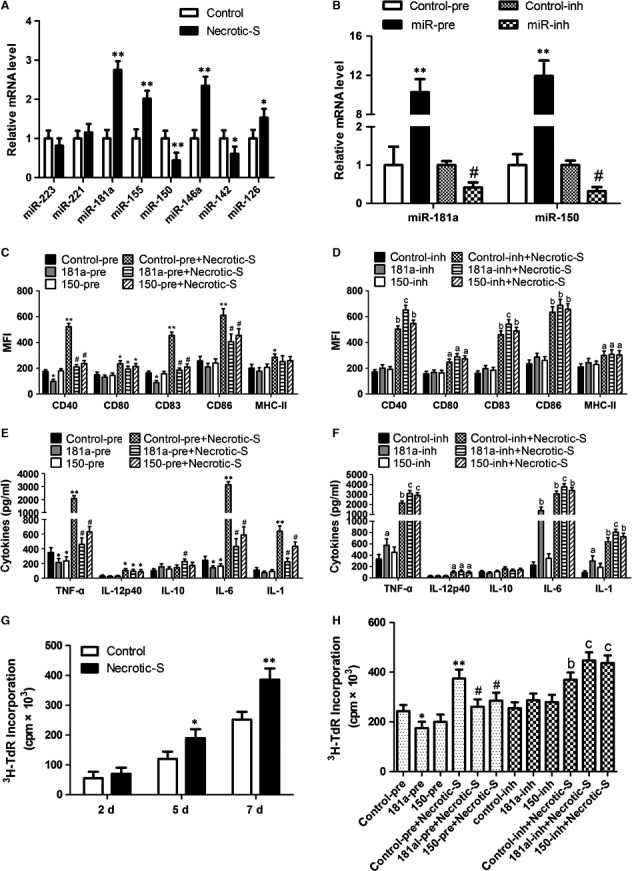
miR‐181a and miR‐150 regulate necrotic cardiomyocyte‐induced DC immune inflammatory responses. (**A**) miR‐223, miR‐221, miR‐181a miR‐155, miR‐150, miR‐146a, miR‐142 and miR‐126 expression assessed by real‐time RT‐PCR in necrotic cardiomyocyte‐treated DCs. Results are shown as relative mRNA expression. **P < 0.01. (**B**) BMDCs were transfected with control‐pre, miR‐181a‐pre, miR‐150‐pre, control‐inh, miR‐181a‐inh, or miR‐150‐inh, and miR‐181a and miR‐150 expression was assessed by real‐time RT‐PCR. **P < 0.01, *versus* the control‐pre group; # *P <* 0.01, *versus* the control‐inh group. These transfected BMDCs were either unstimulated (control group) or stimulated with Necrotic‐S (Necrotic‐S group) for 24 hrs. (**C**,** D**) The expression of cell‐surface CD40, CD80, CD83, CD86 and MHC‐II was determined by flow cytometry of BMDCs. (**E**,** F**) The expression of cytokines including TNF‐α, IL‐12p40, IL‐10, IL‐6, and IL‐1 was analysed by ELISA. *P < 0.05, **P < 0.01, *versus* the control‐pre group; # *P <* 0.01, *versus* the control‐pre+Necrotic‐S group. ^a^ *P <* 0.05, ^b^ *P <* 0.01, *versus* the control‐inh group; ^c^ *P <* 0.05, *versus* the control‐inh+Necrotic‐S group. (**G**) CD4^+^ T lymphocytes were cultured with the unstimulated or Necrotic‐S stimulated BMDCs in triplicate wells at 37°C for 2, 5 or 7 days, pulsed overnight with 1 μCi/well [^3^H] thymidine, harvested, and analysed for T cell proliferation. *P < 0.05, **P < 0.01. (**H**) proliferation of T cells was determined by the incorporation of [^3^H] thymidine into the DNA in the presence of various transfected BMDCs. *P < 0.05, **P < 0.01, *versus* the control‐pre group; # *P <* 0.01, *versus* the control‐pre+Necrotic‐S group. ^a^ *P <* 0.05, ^b^ *P <* 0.01, *versus* the control‐inh group; ^c^ *P <* 0.05, *versus* the control‐inh+Necrotic‐S group.

Previous studies suggested that miR‐223, miR‐221, miR‐181a, miR‐155, miR‐150, miR‐146a, miR‐142 and miR‐126 are related to MI and/or DC maturation/immune inflammatory 19, 20, 21; therefore, the expression of these miRNAs was determined in necrotic cardiomyocyte‐induced BMDCs. Stimulation with Necrotic‐S dramatically up‐regulated the expression of miR‐181a and greatly down‐regulated the expression of miR‐150 in BMDCs (Fig. 2A). To test the potential role of miR‐181a and miR‐150 in necrotic cardiomyocyte‐induced DC immune/inflammatory responses, miR‐181a and miR‐150 were overexpressed using miRNA precursors or inhibited with miRNA inhibitors (Fig. 2B), and the expression of DC maturation markers was analysed by flow cytometry. miR‐181a overexpression down‐regulated CD40 and CD83 and suppressed the necrotic cardiomyocyte‐induced up‐regulation of CD40, CD83 and CD86, whereas it had no effect on CD80 and MHC‐II (Fig. 2C). Inhibition of endogenous miR‐181a by transfection of BMDCs with a miR‐181a inhibitor enhanced necrotic cardiomyocyte‐induced CD40 and CD83 expression (Fig. 2D). Thus, miR‐181a affects DC maturation *in vitro*. miR‐150 overexpression also suppressed the necrotic cardiomyocyte‐induced up‐regulation of CD40, CD83 and CD86 (Fig. 2C); however, inhibition of endogenous miR‐150 had no effect on the expression of DC maturation markers (Fig. 2D), suggesting that miR‐150 is not critical for normal DC maturation.

Analysis of inflammatory cytokine expression showed that miR‐181a or miR‐150 overexpression inhibited TNF‐α and IL‐6 secretion, and attenuated the necrotic cardiomyocyte‐induced secretion of TNF‐α, IL‐6 and IL‐1 (Fig. 2E). miR‐181a increased IL‐10 production in the presence of Necrotic‐S stimulation (Fig. 2E). Inhibition of endogenous miR‐181a further increased TNF‐α, IL‐6, and IL‐1 production and enhanced the effects of Necrotic‐S, whereas inhibition of endogenous miR‐150 only increased TNF‐α in the presence of Necrotic‐S stimulation (Fig. 2F). These findings suggest that miR‐181a and miR‐150 affect the inflammatory response in necrotic cardiomyocyte‐treated DCs.

We next investigated whether Necrotic‐S‐induced DCs affected CD4^+^ T cell proliferation. CD4^+^ T lymphocytes were stimulated with a phytohaemagglutinin (PHA) and then co‐cultured with Necrotic‐S‐induced DCs for 7 days. T cell proliferation was determined using ^3^H‐thymidine incorporation. We found that Necrotic‐S‐induced DCs significantly increased CD4^+^ T cell proliferation at 5 and 7 days after PHA stimulation (Fig. 2G). miR‐181a overexpression significantly inhibited CD4^+^ T cell proliferation, and miR‐181a or miR‐150 overexpression both suppressed Necrotic‐S‐induced CD4^+^ T cell proliferation (Fig. 2H). Inhibition of endogenous miR‐181a or miR‐150 further stimulated CD4^+^ T cell proliferation and enhanced the effects of Necrotic‐S (Fig. 2H).

### miR‐181a and miR‐150 target the JAK‐STAT1/c‐Fos pathway in the Necrotic‐S‐induced DC immune inflammatory response

**Figure 3:**
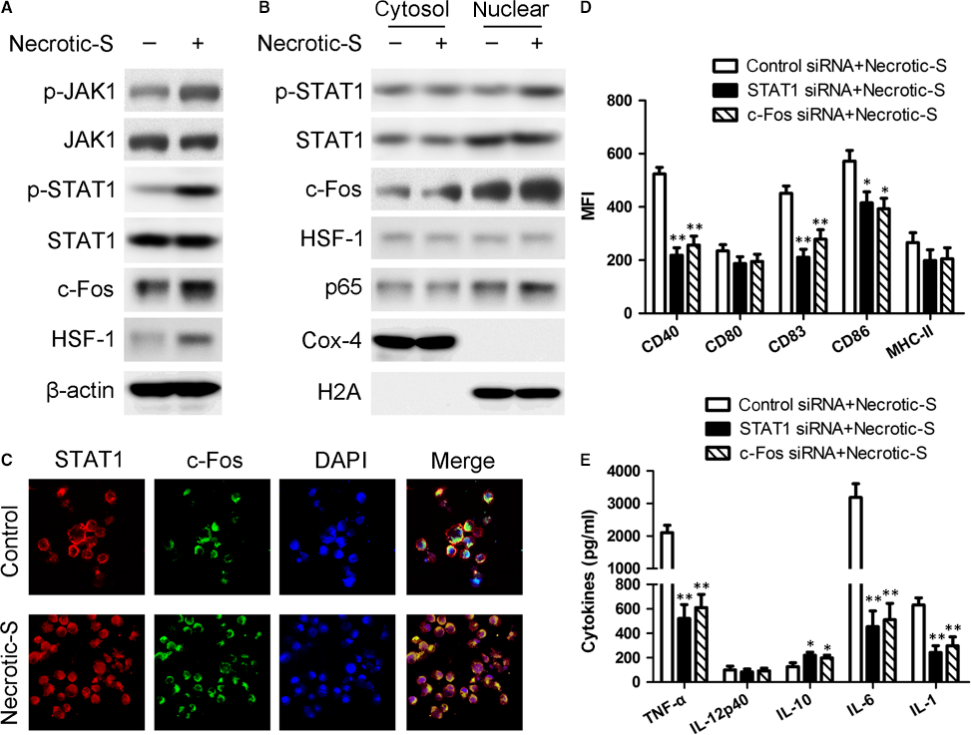
JAK1–STAT1/c‐Fos signalling is involved in necrotic cardiomyocyte‐induced DC immune inflammatory responses. (**A**) Analysis of the JAK1–STAT1 pathway and c‐Fos and HSF‐1 expression in necrotic cardiomyocyte‐induced BMDCs. The total cell lysates from unstimulated or Necrotic‐S stimulated BMDCs were prepared and subjected to Western blotting to analyse the activity of the JAK1/STAT1 pathway and the expression of c‐Fos and HSF‐1. (**B**) Activation and nuclear translocation of STAT1, NF‐κB, AP‐1 and HSF‐1 induced by Necrotic‐S in BMDCs. The cells were harvested and used to prepare cytoplasmic or nuclear extracts, which were subjected to Western blotting to analyse the activation status of STAT1, c‐Fos, HSF‐1 and NF‐κB using antibodies to anti‐phosphotyrosine STAT1, anti‐STAT1, c‐Fos and NF‐κB (p65). Cox‐4 and H2A were used as cytosolic and nuclear internal controls, respectively. (**C**) Immunofluorescence analysis of the nuclear translocation of STAT1 and c‐Fos. DAPI was used as a nuclear stain. (**D and E**) STAT1 and c‐Fos were knocked down by specific siRNA transfection, and the effect on the necrotic‐S‐induced expression of DC maturation markers and inflammatory cytokine production were assessed by flow cytometry and ELISA, respectively.

Signal transducer and activator of transcription 1 (STAT1) is a transcription factor involved in inflammation and DC maturation, and it has been shown to interact with c‐Fos and heat shock factor (HSF)‐1 22, 23. Necrotic‐S‐induced DCs showed significantly increased phosphorylated (p)‐Janus kinase 1 (JAK1) and p‐STAT1 levels, and up‐regulation of the expression of c‐Fos and HSF‐1 (Fig. 3A). Nuclear translocation of STAT1, c‐Fos, HSF‐1 and p65/NF‐κB were analysed by Western blotting. The nuclear translocation profile reflected a strong activation of STAT1, c‐Fos, and p65/NF‐κB, and a weak activation of HSF‐1 (Fig. 3B). The phosphorylation and nuclear translocation of STAT3 and STAT5 was also determined, but no significant changes were observed in Necrotic‐S‐induced DCs compared with control cells (Fig. S2). Immunofluorescence staining showed that Necrotic‐S‐induced DCs promoted the nuclear translocation of STAT1 and c‐Fos (Fig. 3C). SiRNA mediated STAT1 and c‐Fos silencing suppressed the Necrotic‐S‐induced up‐regulation of CD40, CD83, and CD86 (Fig. 3D), and attenuated the Necrotic‐S‐induced secretion of TNF‐α, IL‐6, and IL‐1 and increased IL‐10 production (Fig. 3E). These findings suggested the involvement of JAK1–STAT1/c‐Fos signalling in the Necrotic‐S‐induced DC immune inflammatory response.

**Figure 4:**
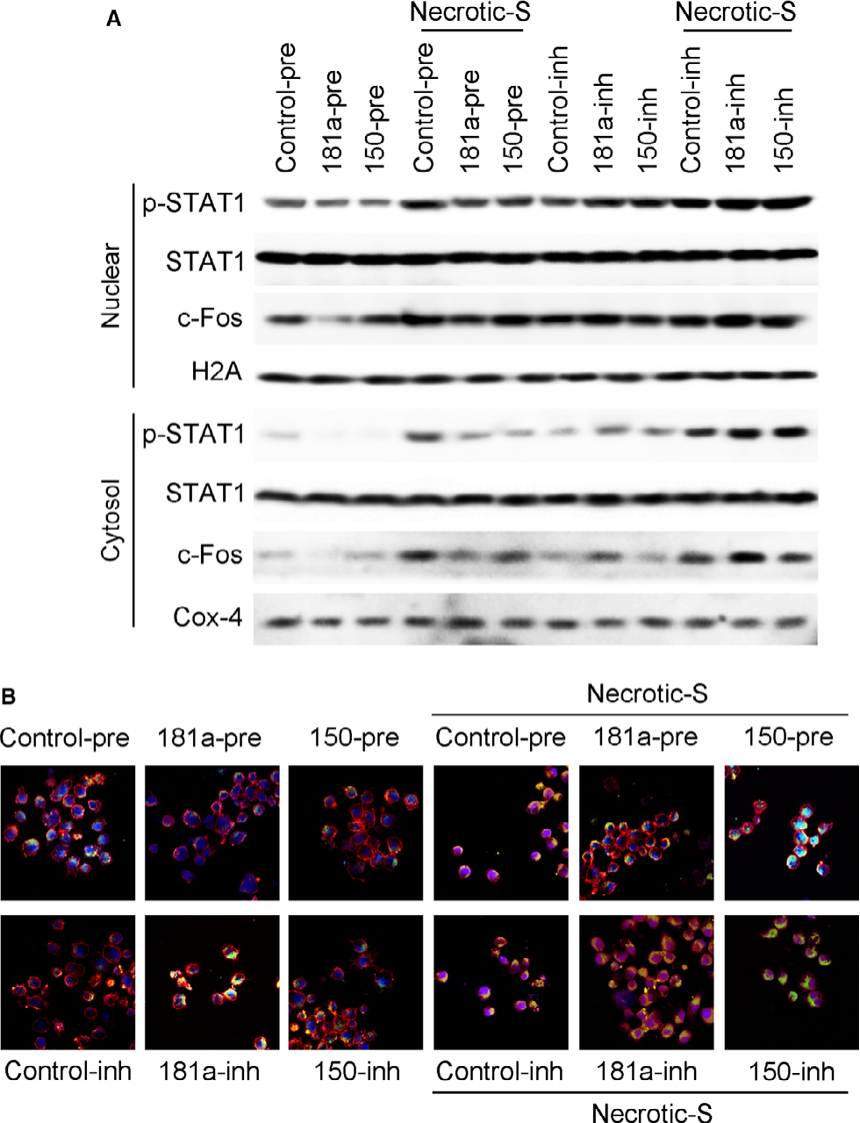
miR‐181a and miR‐150 target STAT1/c‐Fos signalling in necrotic cardiomyocyte‐treated DCs. BMDCs were transfected with control‐pre, miR‐181a‐pre, miR‐150‐pre, control‐inh, miR‐181a‐inh or miR‐150‐inh, and either unstimulated or stimulated with Necrotic‐S for 24 hrs. (**A**) Cytoplasmic and nuclear extracts were then subjected to Western blotting to assess the activation status of STAT1 and c‐Fos. Cox‐4 and H2A were used for cytosolic and nuclear internal controls, respectively. (**B**) Immunofluorescence staining was performed to determine the nuclear translocation of STAT1 and c‐Fos. DAPI was used as a nuclear stain.

**Figure 5:**
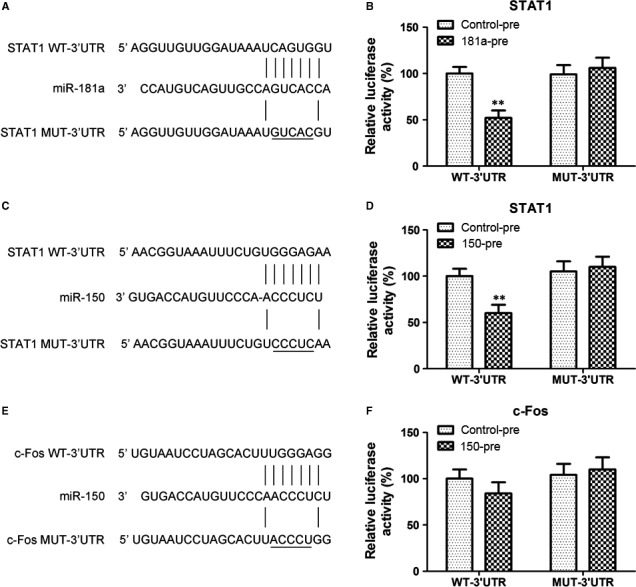
STAT1 is a direct target of miR‐181a and miR‐150. The diagrams represent the sequence alignment of miR‐181a (**A**) and miR‐150 (**C**) and its target sites within the wild‐type (WT) STAT1 3′‐UTR and the related mutant versions (MUT). The mutations made in the STAT1 3′‐UTR are highlighted. HEK293T cells were cotransfected with the STAT1 WT or MUT‐3′‐UTR firefly luciferase reporter plasmids plasmid, together with miR‐181a‐pre (**B**) or miR‐150‐pre (**D**), or control‐pre. Luciferase values were normalized by the pTK‐Renilla control luciferase activity. **P < 0.01, *versus* the WT‐3′‐UTR + control‐pre. (**E**) The sequence of miR‐150 is shown aligned with its predicted target site in the 3′‐UTR of c‐Fos mRNA. The sequence of the mutated 3′‐UTR of c‐Fos mRNA is also shown. (**F**) The reporter gene assay was performed in 293T cells cotransfected with the c‐Fos WT or MUT‐3′‐UTR along with miR‐150‐pre or control‐pre. Luciferase activity was measured and normalized to Renilla luciferase activity. The data are representative from three independent experiments.

Western blot and immunofluorescence analysis showed that miR‐181a overexpression inhibited the nuclear translocation of STAT1 and c‐Fos, and suppressed their Necrotic‐S‐induced nuclear translocation; miR‐150 overexpression only inhibited the nuclear translocation of STAT1, and inhibited the Necrotic‐S‐induced nuclear translocation of STAT1 and c‐Fos (Fig. 4A and B). Inhibition of endogenous miR‐181a further increased the nuclear translocation of STAT1 and c‐Fos and enhanced the effects of Necrotic‐S, whereas inhibition of endogenous miR‐150 promoted the nuclear translocation of STAT1, but only that of c‐Fos in the presence of Necrotic‐S stimulation (Fig. 4). STAT1 and c‐Fos were identified as possible target gene of miR‐181a and miR‐150 by bioinformatics analysis in several databases (*i.e*. Targetscan, PicTar and miRanda). To demonstrate a direct effect of miR‐181a and miR‐150 on STAT1 mRNA, a construct containing the full‐length STAT1 3′‐UTR or the mutant 3′‐UTR in seed region was cotransfected along with miRNA or control‐miRNA precursors into HEK293 cells (Fig. 5A and C). The relative luciferase activities were significantly reduced in cells cotransfected with the full‐length STAT1 3′‐UTR plasmid and miRNA‐pre; and the two miRNAs had no significant effect on the mutated STAT1 3′‐UTR construct (Fig. 5B and D). These results demonstrated a specific and direct effect of miR‐181a and miR‐150 on the STAT1 3′‐UTR. Our previous studies verified that miR‐181a directly targets c‐Fos using luciferase reporter gene assays 12. However, the reporter gene assay showed that the c‐Fos 3′‐UTR was not a direct target of miR‐150 (Fig. 5E and F). These findings indicated that miR‐181a and miR‐150 may affect the inflammatory response by targeting the STAT1/c‐Fos pathway in Necrotic‐S‐treated DCs.

### miR‐181a and miR‐150 modulate cardiomyocyte apoptosis induced by Necrotic‐S treated DCs under hypoxic conditions

**Figure 6:**
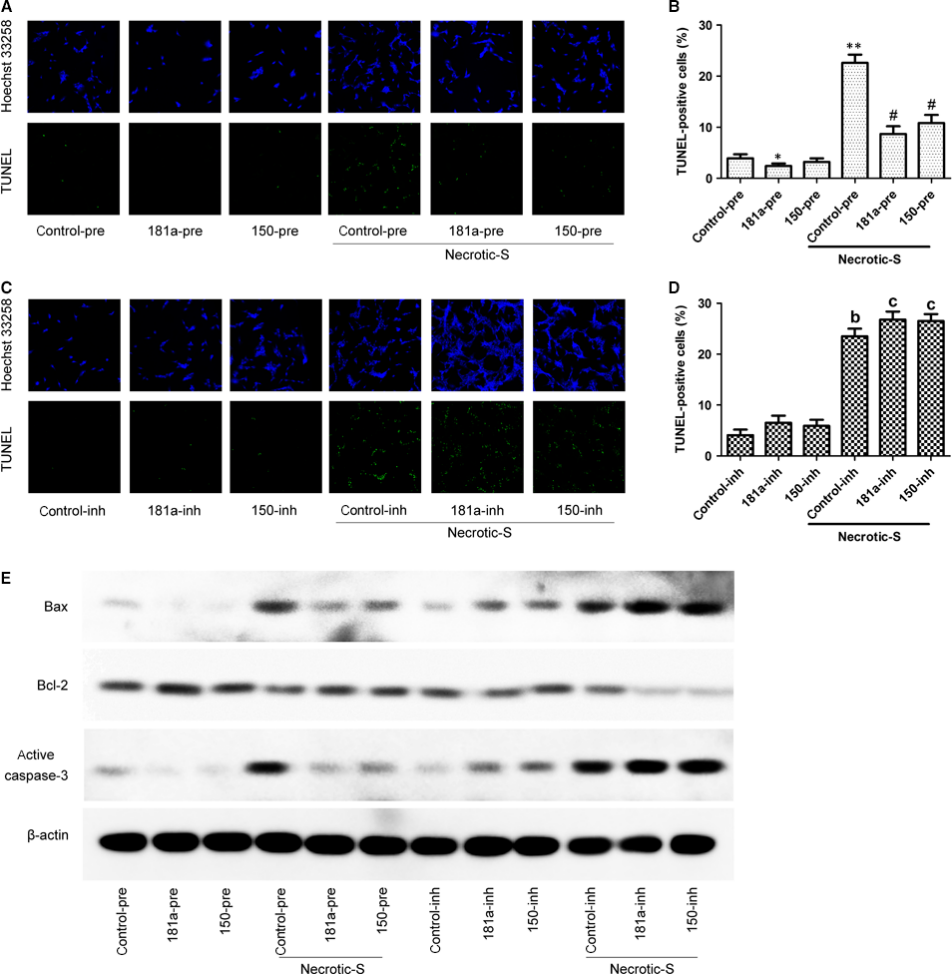
miR‐181a and miR‐150 modulate cardiomyocyte apoptosis induced by necrotic cardiomyocyte‐treated DCs under hypoxic conditions. BMDCs cultured for 7 days were transfected with control‐pre, miR‐181a‐pre, miR‐150‐pre, control‐inh, miR‐181a‐inh or miR‐150‐inh and either unstimulated (control group) or stimulated with Necrotic‐S for 24 hrs. BMDCs suspensions were co‐cultured with primary cardiomyocytes at a 10:1 ratio under hypoxic conditions for 48 hrs and apoptosis was assessed by TUNEL assay. (**A and C**) Representative immunofluorescence microscopy images (magnification, 100×). (**B and D**) The number of TUNEL‐positive cells was counted in six fields per well for each experiment. (**E**) Western blot analysis of Bax, Bcl‐2 and activated caspase‐3. *n* = 3 in each group. Data are expressed as the mean ± S.D. *P < 0.05, **P < 0.01, *versus* the control‐pre group; # *P <* 0.01, *versus* the control‐pre+Necrotic‐S group; ^b^ *P <* 0.01, *versus* the control‐inh group. ^c^ *P <* 0.05, *versus* the control‐inh+Necrotic‐S group.

Hypoxia is a pathophysiological condition frequently occurring in cardiomyocytes. To investigate the effect of miR‐181a and miR‐150 on cell death under hypoxic stress, we cocultured cardiomyocytes with DCs under hypoxic conditions. The TUNEL assay showed that Necrotic‐S‐treated DCs significantly increased cardiomyocyte apoptosis at 48 hrs after hypoxia (Fig 6A and B). The Necrotic‐S‐induced cell death was suppressed by miR‐181a or miR‐150 overexpression (Fig. 6A and B). The inhibition of miR‐181a or miR‐150 significantly enhanced cardiomyocyte apoptosis induced by Necrotic‐S‐treated DCs under hypoxic conditions (Fig. 6C and D). Western blot detection of the expression of apoptosis‐related proteins showed that Necrotic‐S‐treated DCs up‐regulated the pro‐apoptotic proteins Bax and activated caspase‐3 and down‐regulated the anti‐apoptotic protein Bcl‐2, and these effects were suppressed by miR‐181a or miR‐150 overexpression and enhanced by miR‐181a or miR‐150 inhibition (Fig. 6E). Taken together, these results indicated that miR‐181a and miR‐150 suppress cardiomyocyte apoptosis induced by necrotic DCs under conditions of hypoxia.

## Discussion

Accumulating evidence indicates that myocardial damage leads to the activation of innate immunity, which aggravates the injury and prevents remodelling, while at the same time promoting myocardial healing 24. DCs play an important role in the post‐infarction healing process by modulating the activity of monocytes and macrophages 25. In the present study, *in vitro* simulation of the post‐MI microenvironment using Necrotic‐S resulted in the induction of DC maturation, as shown by the up‐regulation of DC maturation markers, and increased the levels of inflammatory cytokines in parallel with the up‐regulation of miR‐180a and down‐regulation of miR‐150. Simulation of the MI microenvironment increased CD4^+^ T cell proliferation, activated the JAK/STAT pathway, promoted the nuclear translocation of c‐Fos and NF‐κB p65 and induced DC apoptosis under conditions of hypoxia, and these effects were suppressed by miR‐181a or miR‐150 overexpression.

Several miRNAs have recently emerged as biomarkers of acute MI (AMI) because of their role in myocardial injury 26. Furthermore, many miRNAs are expressed in immune cells and some of them play a role in DC differentiation and function, most notably miR‐155, miR‐221, let‐7i, miR‐142‐3p and the miR‐148 family 16. The miR‐181 family plays an important role in vascular inflammation and immunity 27. The expression of miR‐181b and miR‐181c in cardiac fibroblasts and cardiomyocytes is associated with cardiac injury and fibrosis as well as the regulation of mitochondrial function 28, 29. miR‐181a regulates inflammatory responses through its role in the modulation of DC function. We previously showed that overexpression of miR‐181a in BMDCs attenuates ox‐LDL‐mediated up‐regulation of CD83 and CD40, inhibits IL‐6 and TNF‐α secretion and increases IL‐10 expression in part through its target c‐Fos, suggesting that miR‐181a represses ox‐LDL induced inflammation 12. miR‐150 was shown to be down‐regulated in a mouse model of AMI and in human samples, and ectopic expression of miR‐150 reduced proinflammatory cytokine production *in vitro* and improved cardiac function, reduced infarction size, reduced inflammatory monocyte invasion and inhibited apoptosis *in vivo* 1. miR‐150 was suggested to exert a cardioprotective effect in AMI by regulating monocyte migration and proinflammatory cytokine production. The levels of circulating miR‐150 are reduced in patients with AMI in association with left ventricular remodelling 30.

In the present study, miR‐181a was up‐regulated and miR‐150 was down‐regulated in BMDCs in response to stimulation with Necrotic‐S, and miR‐181a or miR‐150 overexpression suppressed Necrotic‐S induced up‐regulation of DC maturation markers and inhibited the Necrotic‐S induced secretion of inflammatory cytokines and up‐regulation of CD4^+^ T cell proliferation. However, the expression of miR‐181a and miR‐150 was not affected in miRNA precursor‐overexpressed or miRNA inhibitor‐knockdown cells in pretest (*P* > 0.05, data not shown), suggesting that there may not be regulatory effect on miRNA expression. miRNAs are differentially expressed during the morphological and functional development of DCs 21. Maturation of DC can result in down‐ or up‐regulation of miRNAs. Some miRNAs are constitutively expressed in immature BMDC and then down‐regulated after DC activation, such as miR‐142 and miR‐150 31, 32. Some miRNAs are increased in DC following activation, their expression has a negative effect on DC maturation, such as miR‐146a and miR‐181a 12, 33. This indicates that the up‐regulation of miR‐181a may be an important self‐protection mechanism to compensate the down‐regulation of miRNAs that are constitutively expressed, such as miR‐150. However, additional studies are necessary to clarify this. Further analysis indicated that the effects of Necrotic‐S on DCs were mediated by the activation of the JAK/STAT pathway and c‐Fos and NF‐κB p65 nuclear translocation. JAKs are bound to the cytoplasmic domain of cytokine receptors and are activated and phosphorylated upon binding of cytokines to their receptors, leading to the activation of STATs *via* tyrosine phosphorylation 34. STATs dimerize and are translocated to the nucleus to modulate the expression of target genes. c‐Fos belongs to a group of dimeric transcription factors referred to as activating protein 1 (AP‐1), which are implicated in a wide range of processes such as proliferation, differentiation and apoptosis 35. Several studies have shown that c‐Fos inhibits proinflammatory cytokine production in DCs, and the down‐regulation of c‐Fos by miR‐155, a miRNA involved in the regulation of innate and adaptive immune responses, is required for DC maturation and function. These studies support the present results indicating that the role of miR‐181a and miR‐150 in the regulation of DC inflammatory responses is mediated by the modulation of the JAK/STAT and c‐Fos pathways.

Cardiomyocyte death during MI is attributed not only to necrosis, which is a rapid loss of cellular homoeostasis, plasma membrane rupture and the disruption of cellular organelles leading to an inflammatory response, but also to apoptosis 36. Hypoxia, which is a common condition associated with the ischaemic environment caused by coronary occlusion in MI, triggers cardiomyocyte death. We investigated the roles of miR‐181a and miR‐150 in cardiomyocyte apoptosis induced by hypoxic stress. Our results showed that Necrotic‐S treated DCs significantly increased cardiomyocyte apoptosis at 48 hrs after hypoxia, and this cell death was suppressed by miR‐181a or miR‐150 overexpression. This suggested a protective effect of miR‐181a and miR‐150 in the heart in the MI microenvironment and in association with DC‐mediated inflammatory responses. In previous studies, Bim, a BH3‐only Bcl‐2 family member that is required for apoptosis initiation, was identified as a target of miR‐181a in a rat model of retinal ischaemia–reperfusion 37. miR‐150 plays a cardioprotective role during ischaemic injury by repressing the pro‐apoptotic genes egr2 and p2x7r in cardiomyocytes 38. Liu *et al*. showed that miR‐150 protects the heart from ischaemic injury by inhibiting the migration of inflammatory monocytes in a mouse model of AMI 1. However, the exact mechanism by which these miRNAs modulate apoptosis in cardiomyocytes remains to be elucidated, and future studies should be aimed at identifying relevant targets of miR‐181a and miR‐150 involved in the response to hypoxia and inflammation.

In summary, the present study showed that miR‐181a and miR‐150 play a protective role in DC inflammatory responses in the context of the MI microenvironment *via* a mechanism involving JAK1/STAT1/c‐Fos signalling and inhibit cardiomyocyte apoptosis under conditions of hypoxia. Our findings suggest potential biomarkers and therapeutic targets in myocardial injury and remodelling associated with MI.

## Conflict of interest

None.

## Supporting information


**Figure S1** Flow cytometry analysis of cardiomyocytes necrosis.

**Figure S2** The phosphorylation and nuclear translocation of STAT3 and STAT5 in necrotic cardiomyocyte‐induced BMDCs.
