# Supplementary figures and images for: miR‐181a and miR‐150 regulate dendritic cell immune inflammatory responses and cardiomyocyte apoptosis via targeting JAK1–STAT1/c‐Fos pathway
Source: J Cell Mol Med. 2017 Jun 9;21(11):2884–95. doi: 10.1111/jcmm.13201 (PMC5661264; doi:10.1111/jcmm.13201)

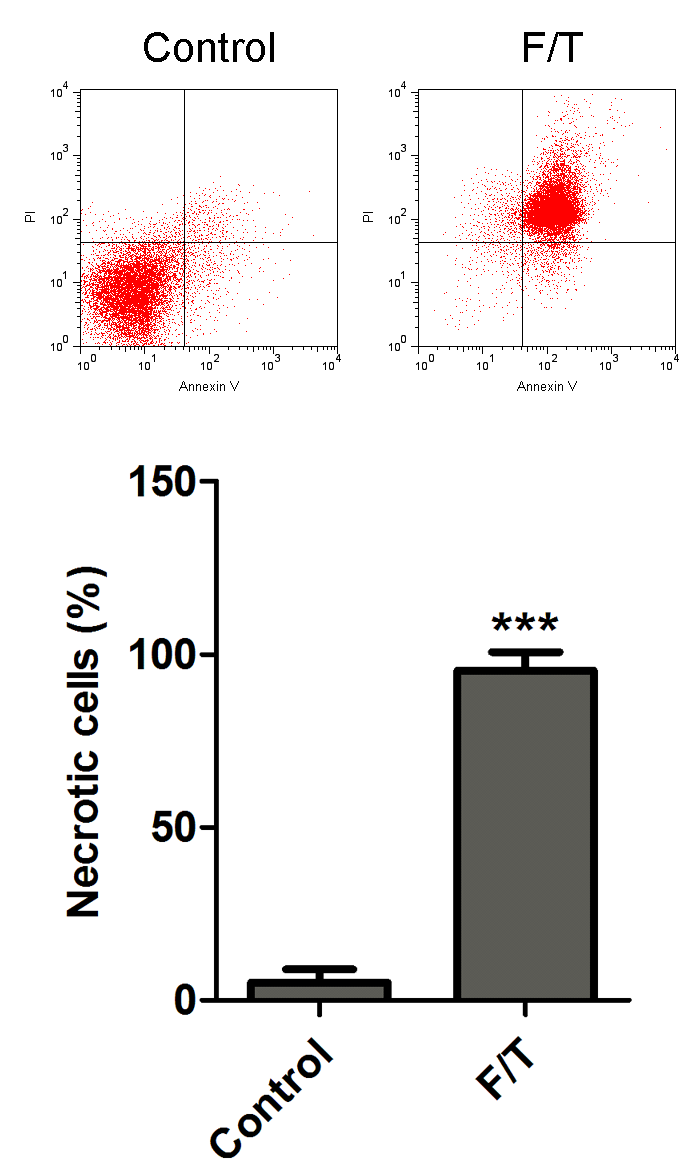

Supplement: Supplementary file 1 — Figure S1 Flow cytometry analysis of cardiomyocytes necrosis. [file JCMM-21-2884-s001.tif]

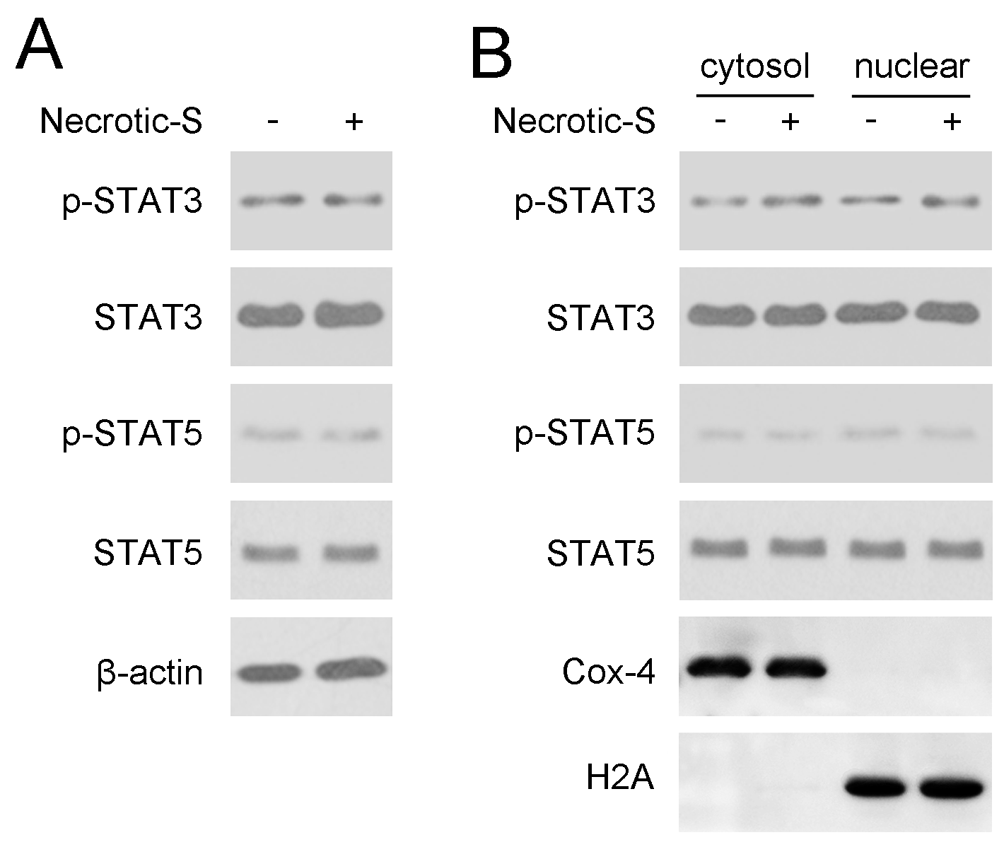

Supplement: Supplementary file 2 — Figure S2 The phosphorylation and nuclear translocation of STAT3 and STAT5 in necrotic cardiomyocyte‐induced BMDCs. [file JCMM-21-2884-s002.tif]
